# Factors associated with the level of knowledge about CAD/CAM use among Peruvian dental students

**DOI:** 10.1038/s41598-026-50270-2

**Published:** 2026-04-29

**Authors:** Angela Yataco-Saco, Frank Cotrina-Ruiz, Leonor Castro-Ramirez, José Huamani-Echaccaya, Denisse Turpo-Claudio, Marysela Ladera-Castañeda, César Cayo-Rojas

**Affiliations:** School of Stomatology, Universidad Privada San Juan Bautista, Lima, Peru

**Keywords:** Computer-aided design, Cross-sectional studies, Health knowledge, Students, dental, Technology, dental

## Abstract

The use of computer-aided design/computer-aided manufacturing (CAD/CAM) enables a faster and more flexible workflow, reducing costs and work time. This study aimed to determine factors associated with the level of knowledge about CAD/CAM use among Peruvian dental students. This analytical, cross-sectional, observational study assessed 301 Peruvian dental students between March and April 2025 using a CAD/CAM knowledge questionnaire. A multivariable Poisson regression model with robust variance was fitted to estimate adjusted prevalence ratios (APR) for the association between knowledge level and sex, age, year of study, marital status, place of origin, occupation, and prior CAD/CAM training. Statistical significance was set at *p* < 0.05. When summarized using prespecified descriptive score groupings, 14.0% (95% CI: 10.0–17.9%) of students fell into the poor range, 66.4% (95% CI 61.1–71.8%) into the fair range, and 19.6% (95% CI 15.1–24.1%) into the good range regarding CAD/CAM use. Item-level findings were examined for descriptive and exploratory purposes and suggested frequent misconceptions, particularly concerning the complete elimination of physical impressions and the materials compatible with the system. In the adjusted analysis, third-year students showed a higher prevalence of belonging to the poor score grouping for CAD/CAM knowledge than fifth-year students (APR = 2.54; 95% CI 1.27–5.12). No significant associations were observed for age group, sex, marital status, place of origin, occupation, or prior CAD/CAM training. Dental students showed relevant knowledge gaps regarding CAD/CAM, with frequent misconceptions and a predominance of scores in the fair descriptive range. Third-year students showed a higher prevalence of belonging to the poor score grouping for CAD/CAM knowledge than fifth-year students, whereas demographic variables and prior training were not significantly associated; however, these findings should be interpreted cautiously given the preliminary measurement evidence and because the score groupings were used solely as operational, descriptive categories within this study rather than as validated or competency-based thresholds.

**Supplementary Information:**

The online version contains supplementary material available at 10.1038/s41598-026-50270-2.

## Introduction

High-quality learning in dentistry requires educational activities tailored to the type and duration of clinical practice^1^. Practical training remains essential for acquiring clinical procedures^1^. Nevertheless, surveys among dental undergraduates report variable awareness and limited curricular exposure to CAD/CAM, suggesting uneven competency development^2^. However, the digital evolution of dentistry has enabled fully digital workflows (e.g., intraoral scanning, digital design, and CAD/CAM fabrication), which require specific competencies during training^3^. Consequently, dental education should strengthen its approach to preparing students to meet these emerging digital demands^4^.

The integration of CAD/CAM into teaching and learning processes offers students opportunities to expand their experience in prosthodontic procedures and other areas of dentistry^5^. Survey-based evidence indicates growing interest and support for incorporating CAD/CAM into undergraduate training^5–7^. Thus, digital technology is increasingly viewed as essential to modern dentistry and is expected to further transform practice in the future^8^. Nonetheless, evidence suggests that CAD/CAM is not yet consistently embedded in undergraduate curricula and that training may remain predominantly theoretical in some settings, with limited supervised hands-on opportunities^2,8–11^.

Evidence suggests that students generally perceive digital dentistry positively and support its inclusion in training, while also highlighting implementation barriers and the need for structured educational approaches^10,11^. Therefore, ensuring appropriate CAD/CAM training during undergraduate education is important to keep students current with evolving techniques and may ultimately contribute to improved clinical practice^12^. Given the advantages of digital dentistry and the role of CAD/CAM in contemporary workflows, it is pertinent to assess students’ knowledge of this technology^13–15^. International evidence indicates substantial variability in how CAD/CAM is integrated into curricula, and context-specific data remain limited, particularly in Latin America, including Peru, which underscores the need for targeted curricular planning in this setting^2,4,5,8,11,16^. Moreover, identifying sociodemographic and academic factors associated with CAD/CAM knowledge may help institutions prioritize early interventions and tailor training strategies to student needs^2,5,8^. To our knowledge, evidence addressing associated factors in this context remains scarce.

Accordingly, this study aimed to determine the factors associated with the level of knowledge about CAD/CAM use among Peruvian dental students. The null hypothesis posited that there are no factors associated with the level of knowledge about CAD/CAM use among Peruvian dental students.

## Methods

### Study design

This analytical, cross-sectional observational study was reported in accordance with the Strengthening the Reporting of Observational Studies in Epidemiology (STROBE) statement for observational studies^17^. The study was conducted from March to April 2025 at the Universidad Privada San Juan Bautista (UPSJB), Peru, including the main campus in Lima and the branch campus in Ica.

### Population and participant selection

The total population consisted of 332 dental students—185 from Lima and 147 from Ica. By year of study, Lima had 32 third-year, 86 fourth-year, and 67 fifth-year students; Ica had 26 third-year, 68 fourth-year, and 53 fifth-year students. The sample size was calculated using the finite-population formula for a single proportion in EPIDAT 4.2, assuming a 95% confidence level, an expected proportion of 50% (to maximize the sample size), and 5% absolute precision, yielding a minimum required size of 226. Given this, the entire study population was invited. Accordingly, after applying the selection criteria, the final target population was N = 301 students: Lima, 30 third-year, 86 fourth-year, and 67 fifth-year; Ica, 11 third-year, 61 fourth-year, and 46 fifth-year.

Inclusion criteria: dental students enrolled in the 3rd to 5th academic years at UPSJB, aged ≥ 18 years, who provided informed consent.

Exclusion criteria: failure to complete the questionnaire or interruption of the academic term during the study period.

### Variables

The dependent variable was questionnaire-assessed declarative knowledge regarding CAD/CAM use, as measured by a de novo questionnaire developed for this study^2^. Accordingly, the outcome should be interpreted as reflecting conceptual understanding of CAD/CAM-related principles and applications rather than as an objective or externally validated measure of CAD/CAM competence, actual clinical competence, or hands-on proficiency with CAD/CAM systems. Independent variables were age^10,11^, sex^11^, year of study^2,10,11^, marital status, place of origin, occupation, and prior CAD/CAM training.

### Instrument administration

Data were collected using a de novo questionnaire developed by the authors. Item generation was informed by a focused review of the literature on CAD/CAM use in dentistry, including prior surveys^2,5^, and the items were aligned with domains commonly reported in the field. The instrument was written with no verbatim reproduction from previously published questionnaires and was administered in Spanish, tailored to the Peruvian undergraduate context [See Supplementary material]. Content validity was assessed by three experienced judges (one Oral Rehabilitation specialist and two dental researchers), who evaluated pertinence, objectivity, relevance, currency (i.e., the extent to which the items reflect up-to-date CAD/CAM concepts and workflows), and clarity, yielding Aiken’s V = 0.93 (95% CI 0.90–0.96), considered acceptable. Internal consistency was estimated using Cronbach’s alpha, α = 0.67 (95% CI 0.51–0.79), indicating moderate internal consistency.

The factorial validity of the instrument, scored dichotomously (correct/incorrect), supported an essentially unidimensional structure. The tetrachoric correlation matrix for the 10 items yielded a determinant of 0.11, indicating absence of problematic multicollinearity and suitability for factor analysis. An exploratory factor analysis using unweighted least squares extraction with a one-factor solution showed substantial loadings for items Q8 (0.85), Q3 (0.80), Q9 (0.62), and Q5 (0.45), indicating a core of items that are strongly saturated by the same latent construct, as well as good precision of the factor scores (correlation between factor scores and the factor = 0.92; R^2^ = 0.85). Taken together, the correlation pattern, the determinant of the matrix, and the configuration of factor loadings supported the retention of a single latent factor and the use of a unidimensional total score to represent the construct assessed. These findings were interpreted as preliminary structural evidence supporting the operational use of a total score in this study, rather than as evidence of full construct validation.

For test–retest reliability, the questionnaire was administered to 40 dental students at two time points seven days apart, with the item order altered at retest to minimize recall bias. Total scores were non-normally distributed (Kolmogorov–Smirnov test, *p* < 0.05); therefore, Spearman’s correlation was used, yielding ρ = 0.83 (95% CI 0.68–0.93), which was interpreted as acceptable preliminary evidence of score stability over time.

The instrument comprised two sections. Section “General information” in Supplementary material collected sociodemographic information (age, sex, year of study, marital status, place of origin, occupation, and prior training). Section “Questionnaire on knowledge of CAD/CAM use” in Supplementary material contained 10 items assessing knowledge about CAD/CAM use. Items Q1–Q9 offered Yes / No / Don’t know response options, and Q10 was open-ended. For Q10 (open-ended), responses were coded as correct if students explicitly referred to “computer-aided design” and “computer-aided manufacturing” in dentistry (or clear equivalents, such as “digital/computer-assisted design” and “computer-assisted manufacturing/fabrication”). Two investigators (AYS and FCR) independently coded all Q10 responses, and any discrepancies were resolved by consensus. Each correct answer scored 1 and each incorrect answer 0. The total score (0–10) was summarized descriptively using three prespecified score groupings: poor (0–3), fair (4–6), and good (7–10). These cut-off points were defined a priori solely for descriptive purposes in this study and should not be interpreted as validated thresholds, competency standards, or empirically derived knowledge levels. The non-normal distribution of total scores (Kolmogorov–Smirnov test, *p* < 0.05) informed the choice of analytical methods, but not the substantive interpretation of the score groupings.

For modelling associated factors, the total score was dichotomized for analytic purposes as poor = 1 versus fair/good = 0. This dichotomization was used as an operational strategy within this sample and should not be interpreted as a validated diagnostic or competency threshold. Classification consistency for this operational dichotomization was examined using Livingston’s K^2^ coefficient, which yielded 0.83 and was considered acceptable.

### Procedure

The questionnaires were distributed in person by two investigators (A.Y.S. and F.C.R.), who were neither professors nor university administrative staff at the institution where the study was conducted. To minimize social desirability and interviewer bias, the investigators followed a standardized administration script, emphasized anonymity and that responses would not affect academic standing, and avoided providing prompts, feedback, or interpretive guidance while participants completed the questionnaire. The first page of the questionnaire contained the informed-consent statement, which clearly described the study objective, voluntary participation, data confidentiality, and the risk–benefit balance. Students who provided written informed consent were permitted to proceed with completion of the questionnaire. Participants were informed that they could withdraw at any time if they felt uncomfortable responding. The institutional email addresses of the principal investigator and the chair of the ethics committee were provided to address any questions or concerns. All data were stored on a password-protected portable digital device accessible only to the investigators. To preserve confidentiality, paper forms were destroyed after digitization. No incentives were offered for participation. Upon completion of the study, results were emailed to participants who requested them. Data collection took place from 1 March to 30 April 2025.

### Data analysis

Data were analysed using SPSS, version 24.0 (IBM Corp., Armonk, NY, USA) and Stata, version 18.0 (StataCorp LLC, College Station, TX, USA). Categorical variables were summarized with frequency tables; continuous variables with mean, median, and standard deviation. For bivariate analyses of categorical variables, Pearson’s chi-squared test was used, and Fisher’s exact test was applied when expected cell counts were < 5. Somers’ D test was used to assess associations between nominal and ordinal variables. Variables were selected for the adjusted model based on crude associations, and those with *p* < 0.20 in the crude Poisson models were retained for multivariable adjustment. Multicollinearity was assessed using variance inflation factors (VIF), with values below 5 considered indicative of no concerning collinearity. For the multivariable analysis, a Poisson regression model with robust variance was fitted to estimate adjusted prevalence ratios (APR). Statistical significance was set at *p* < 0.05. Because the outcome was derived from a study-specific de novo questionnaire with preliminary psychometric evidence, associations from regression models were interpreted as pertaining to questionnaire-assessed knowledge rather than objectively verified CAD/CAM competence.

### Ethical considerations

The Institutional Research Ethics Committee of the Universidad Privada San Juan Bautista approved the conduct of this study (approval letter No. 278–2025-CIEI-UPSJB, 31 January 2025). The study adhered to the bioethical principles of the Declaration of Helsinki—respect, autonomy, non-maleficence, and confidentiality^18^. Written, voluntary informed consent was obtained on the first page of the questionnaire.

## Results

Among the 301 dental students surveyed, the mean age was 24.0 ± 4.3 years, and 50.8% were aged 22 years or younger. Women comprised 63.8% of the sample, and 48.8% were in the fourth year of study. Most participants were single (92.7%), and 60.8% were from the capital. Additionally, 55.8% reported working while studying. Overall, 77.1% had no prior training in CAD/CAM (Table 1).

**Table 1:** Sociodemographic characteristics of dental students.

Variables	Categories	Frequency	Percentage
Age group	≤ 22 years	153	50.8
	> 22 years	148	49.2
Sex	Male	109	36.2
	Female	192	63.8
Year of study	Third-year	41	13.6
	Fourth-year	147	48.8
	Fifth-year	113	37.5
Marital status	Single	279	92.7
	Married or cohabiting	22	7.3
Place of origin	Capital	183	60.8
	Province	118	39.2
Occupation	Working and studying	168	55.8
	Studying only	133	44.2
Prior CAD/CAM training	Yes	69	22.9
	No	232	77.1
	Mean	Median	SD
Age	24.0	22.0	4.3

SD, standard deviation.

**Fig. 1:**
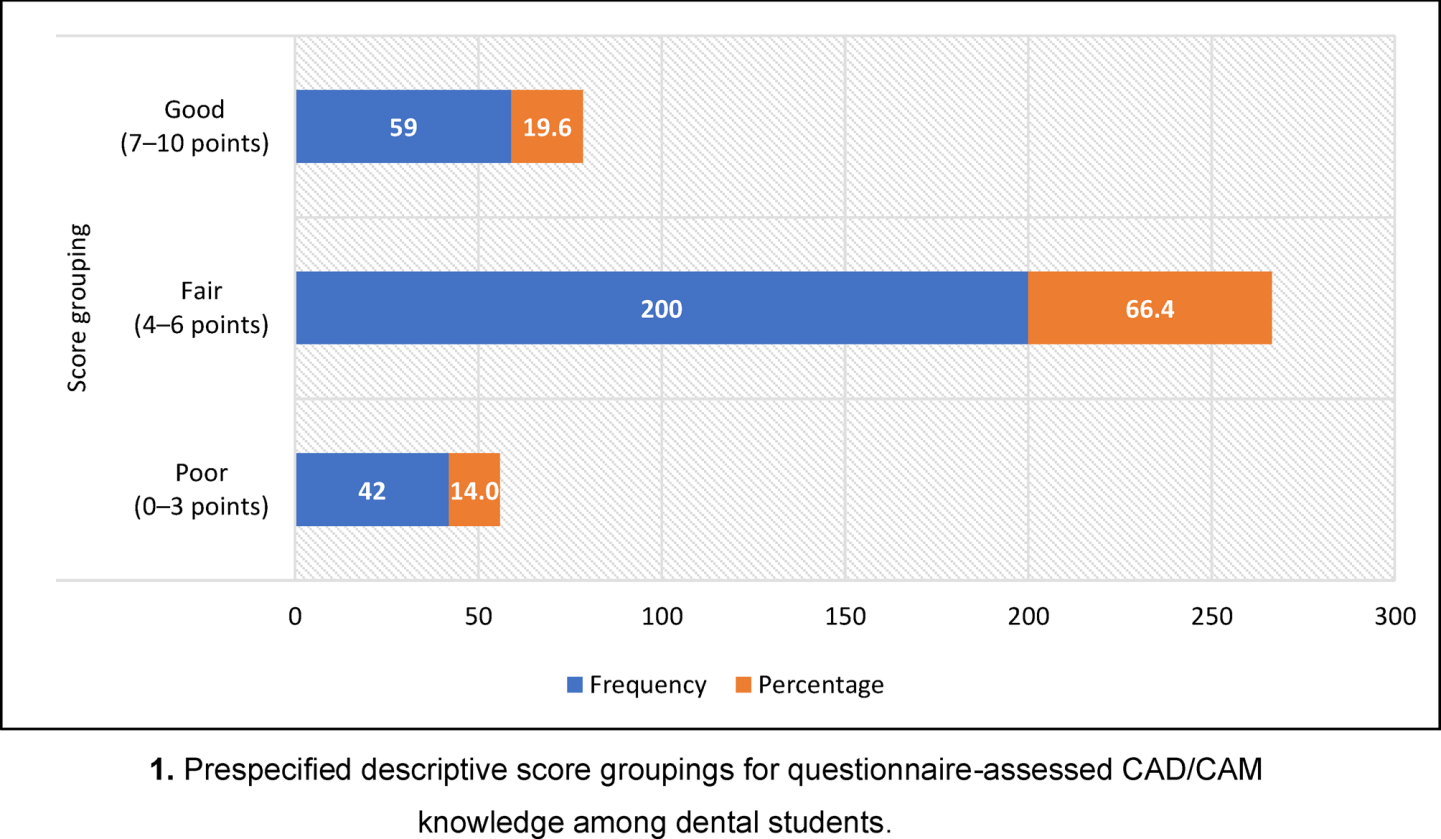
Prespecified descriptive score groupings for questionnaire-assessed CAD/CAM knowledge among dental students.

When summarized using the prespecified descriptive score groupings, 14.0% (95% CI 10.0–17.9) of students fell into the poor range, 66.4% (95% CI 61.1–71.8) into the fair range, and 19.6% (95% CI 15.1–24.1) into the good range (Table 2 and Fig. 1). These groupings are presented for descriptive purposes and should not be interpreted as validated knowledge thresholds.

**Table 2:** Distribution of prespecified descriptive score groupings for questionnaire-assessed CAD/CAM knowledge.

Prespecified descriptive score grouping	N	f	%	95% CI
Poor	301	42	14.0	(10.0–17.9)
Fair	301	200	66.4	(61.1–71.8)
Good	301	59	19.6	(15.1–24.1)

n, sample size; f, frequency; p, proportion (%); 95% CI, 95% confidence interval.

For descriptive and exploratory purposes, item-level comparisons suggested that year of study was associated with response patterns for Q1, Q3, and Q4 (*p* = 0.008, *p* = 0.027, and *p* = 0.030, respectively), and prior CAD/CAM training with Q6 (*p* = 0.046). These comparisons should be interpreted cautiously and not as confirmatory evidence of item-specific differences. Independently of these exploratory comparisons, a large proportion of students did not know that CAD/CAM in dentistry does not completely eliminate the need for physical impressions, were unaware that metals can be used in addition to zirconia discs, and were unable to define CAD/CAM (Table 3).

**Table 3:** Knowledge regarding CAD/CAM use associated with sociodemographic variables among dental students.

Questionnaire	Responses	f (%)	Age group	Sex	Year of Study	Marital status	Place of origin	Occupation	Prior CAD/CAM training
			p*	p*	p*	p*	p*	p*	p*
Q1. Is it possible to design and fabricate an infallible dental prosthesis using CAD/CAM without requiring any intraoral adjustment?	Incorrect	192 (63.8)	0.564	0.528	0.008*	0.349	0.671	0.969	0.571
	Correct	109 (36.2)							
Q2. Does the use of CAD/CAM in dentistry completely eliminate the need for physical impressions of the patient’s mouth?	Incorrect	224 (74.4)	0.407	0.975	0.817	0.750	0.389	0.599	0.405
	Correct	77 (25.6)							
Q3. Is prior instruction indispensable to ensure the correct use of a CAD/CAM system?	Incorrect	20 (6.6)	0.939	0.550	0.027*	0.171	0.383	0.141	0.787
	Correct	281 (93.4)							
Q4. In addition to zirconia discs, can metal be used as a material with CAD/CAM technology?	Incorrect	261 (86.7)	0.571	0.214	0.030*	0.511	0.200	0.911	0.637
	Correct	40 (13.3)							
Q5. Can a complete denture be fabricated using CAD/CAM technology?	Incorrect	76 (25.2)	0.154	0.337	0.267	0.070	0.092	0.086	0.654
	Correct	225 (74.8)							
Q6. Can a post and core be fabricated using CAD/CAM technology?	Incorrect	171 (56.8)	0.001	0.985	0.448	0.503	0.058	0.202	0.046*
	Correct	130 (43.2)							
Q7. Does three-dimensional (3D) printing preclude the use of new materials in dentistry?	Incorrect	202 (67.1)	0.868	0.467	0.192	0.127	0.338	0.854	0.281
	Correct	99 (32.9)							
Q8. Is CAD/CAM technology more accurate than conventional procedures?	Incorrect	37 (12.3)	0.324	0.214	0.314	0.741	0.588	0.349	0.536
	Correct	264 (87.7)							
Q9. Would a restoration fabricated using CAD/CAM be faster than with the conventional method?	Incorrect	64 (21.3)	0.881	0.070	0.521	0.430	0.753	0.291	0.656
	Correct	237 (78.7)							
Q10. Define the term CAD/CAM	Incorrect	222 (73.8)	0.126	0.231	0.072	0.372	0.177	0.581	0.368
	Correct	79 (26.2)							

Based on Pearson’s chi-squared test; Fisher’s exact test was used when expected cell counts were < 5 (*p* < 0.05, significant association).

Using the prespecified descriptive score groupings, bivariable analyses did not identify statistically significant associations between sociodemographic variables and the grouped CAD/CAM knowledge score (*p* > 0.05) (Table 4).

**Table 4:** Bivariable analysis of prespecified descriptive CAD/CAM knowledge score groupings according to sociodemographic variables.

Variable	Categories	Level of knowledge			p*
		Poor	Fair	Good	
Age group	≤ 22 years	23 (7.6)	100 (33.2)	30 (10.0)	0.752
	> 22 years	19 (6.3)	100 (33.2)	29 (9.6)	
Sex	Male	13 (4.3)	71 (23.6)	25 (8.3)	0.223
	Female	29 (9.6)	129 (42.9)	34 (11.3)	
Year of study	Third-year	12 (4.0)	23 (7.6)	6 (2.0)	0.051
	Fourth-year	17 (5.6)	103 (34.2)	27 (9.0)	
	Fifth-year	13 (4.3)	74 (24.6)	26 (8.6)	
Marital status	Single	39 (13.0)	185 (61.5)	55 (18.3)	0.920
	Married or cohabiting	3 (1.0)	15 (5.0)	4 (1.3)	
Place of origin	Capital	25 (8.3)	116 (38.5)	42 (14.0)	0.151
	Province	17 (5.6)	84 (27.9)	17 (5.6)	
Occupation	Working and studying	23 (7.6)	108 (35.9)	37 (12.3)	0.348
	Studying only	19 (6.3)	92 (30.6)	22 (7.3)	
Prior CAD/CAM training	Yes	11 (3.7)	44 (14.6)	14 (4.7)	0.848
	No	31 (10.3)	156 (51.8)	45 (15.0)	

Based on Somers’ D test (*p* < 0.05, significant association).

In the crude Poisson regression model with robust variance, using prevalence ratios (PR), the outcome of interest was membership in the poor score grouping for CAD/CAM knowledge among dental students. Independent variables were age group, sex, year of study, marital status, place of origin, occupation, and prior CAD/CAM training. In the adjusted model, third-year students showed a higher prevalence of belonging to the poor CAD/CAM knowledge score grouping than fifth-year students (APR = 2.54; 95% CI 1.27–5.12). No statistically significant associations were observed for age group, sex, marital status, place of origin, occupation, or prior CAD/CAM training (*p* > 0.05) (Table 5).

**Table 5:** Multivariable analysis of the poor CAD/CAM knowledge score grouping according to sociodemographic variables.

Variables	Categories	VIF	Crude model						Adjustment model				
			β	PR	95% CI		p*		β	APR	95% CI		p**
					LL	UL					LL	UL	
Age group	≤ 22 years	1.29	0.07	1.07	0.53	2.17	0.854						
	> 22 years			*Ref*									
Sex	Male	1.04	-0.22	0.80	0.45	1.43	0.451						
	Female			*Ref*									
Year of study	Third-year	1.25	0.97	2.62	1.25	5.53	0.011*		0.93	2.54	1.27	5.12	0.009**
	Fourth-year	1.21	-0.02	0.98	0.49	1.98	0.964		0.01	1.01	0.51	1.98	0.988
	Fifth-year			*Ref*						*Ref*			
Marital status	Single	1.12	0.02	1.02	0.33	3.14	0.969						
	Married or cohabiting			*Ref*									
Place of origin	Capital	1.09	-0.16	0.86	0.48	1.54	0.604						
	Province			*Ref*									
Occupation	Working and studying	1.22	0.13	1.14	0.60	2.20	0.685						
	Studying only			*Ref*									
Prior CAD/CAM training	Yes	1.05	0.22	1.25	0.66	2.36	0.497						
	No			*Ref*									

*Crude model of prevalence ratios; variables with *p* < 0.20 were selected for inclusion in the adjusted model.

**Adjusted multiple regression model (*p < 0.05, significant association).

APR, adjusted prevalence ratio from a Poisson regression model with robust variance; β, regression coefficient (log PR); 95% CI, 95% confidence interval; LI, lower limit; LS, upper limit; VIF, variance inflation factor.

## Discussion

Digital dentistry has expanded rapidly, with digital workflows enabling faster, simpler, and more efficient clinical processes^16^. In parallel, curriculum updating has become a strategic priority as dental programs transition toward the digital era^19,20^. Accordingly, this study aimed to identify factors associated with CAD/CAM knowledge among Peruvian dental students. Within the limits of the measurement approach and the operational score grouping used in the analyses, third-year students showed a higher prevalence of belonging to the poor score grouping for CAD/CAM knowledge than fifth-year students; therefore, the null hypothesis was rejected.

When the total score was summarized using the prespecified descriptive score groupings, most students fell into the fair range (66.4%), whereas smaller proportions fell into the good (19.6%) and poor (14.0%) ranges. This pattern was broadly consistent with Hall et al.^19^ in Egypt, but differed from findings reported in Pakistan, where lower knowledge predominated^21^, and from those in India, where good knowledge of CAD/CAM indications was more frequently observed^22^. These variations may reflect differences in curricular exposure, as the formal integration of CAD/CAM into undergraduate training has been associated with greater familiarity with its concepts and applications^14,15,23^. In the present study, the predominance of scores in the fair range may be related to the large proportion of fifth-year students, who are more likely to have been exposed to digital technologies through clinical training, even in the absence of formal curricular integration^2^.

In line with Hall et al.^19^, this study found that students in more advanced stages of training tended to show higher CAD/CAM knowledge scores. Similarly, Alhamed et al.^24^ reported progressive gains in knowledge over the years of study, likely due to greater exposure and hands-on experience. These findings suggest that CAD/CAM-related content may need to be introduced earlier and reinforced more consistently throughout the dental curriculum to support progressive knowledge development before graduation.

By contrast, prior CAD/CAM training showed no significant association with CAD/CAM knowledge, in contrast to Alhamed et al.^24^, who found that students exposed to video demonstrations, conference presentations, hands-on workshops, or small-group sessions had higher knowledge scores than those without such exposure. Similarly, Schwindling et al.^23^ indicated that small-group practical courses are particularly effective for teaching digital restoration design. This discrepancy may reflect differences in the quality, duration, content, and timing of the training received, as well as the absence of formal curricular integration. From an educational perspective, these findings suggest that isolated training, without structured practical follow-up, may be insufficient to produce measurable learning gains.

A salient finding was that most students were unaware that CAD/CAM does not completely obviate the need for physical impressions, were unfamiliar with the use of metals in addition to zirconia discs, and were unable to define the term adequately. This pattern aligns with Palanisamy et al.^2^ and Gad et al.^25^, who identified superficial knowledge regarding materials and clinical applications. In contexts where CAD/CAM is not addressed through a structured curricular component, knowledge may be acquired in a partial and predominantly theoretical manner, with limited opportunities for supervised hands-on application^2,25^. This may explain why students encounter CAD/CAM as a set of fragmented “rules” rather than as an integrated workflow grounded in clinical indications, material science, and procedural constraints. When exposure is primarily theoretical and not reinforced through repeated supervised practice, students may overgeneralize simplified messages (e.g., “digital replaces conventional”) and fail to differentiate between intraoral scanning, laboratory scanning, and the continued need for conventional impressions in specific clinical scenarios. Similarly, limited structured teaching on restorative materials and manufacturing pathways may lead to an overly narrow association of CAD/CAM with zirconia only, despite the broader range of machinable alloys and ceramics. Accordingly, curricula should incorporate up-to-date digital dentistry content and provide supervised practice, ideally through case-based learning and simulation with formative feedback, to directly target common misconceptions and support competency development^8,14,23,24^. Future studies should replicate this assessment across universities using objective, competency-based measures to better capture CAD/CAM skills acquired during training. In addition, it would be valuable to evaluate whether and how virtual or blended instruction influences competency acquisition and subsequent clinical adoption.

Regarding the multivariable regression model, third-year students showed a higher prevalence of belonging to the poor score grouping for CAD/CAM knowledge than fifth-year students. This finding should be interpreted cautiously, particularly because CAD/CAM is not formally included in the evaluated university’s curriculum. One possible explanation is that senior students may be more likely to seek extracurricular learning opportunities on their own initiative, motivated by interest in technologies that optimize laboratory and clinical workflows. Greater exposure to digital innovations through social media, specialised sources, and clinical training may also increase familiarity with the practical relevance of these tools. However, residual confounding cannot be excluded, as unmeasured factors such as informal training, extracurricular courses, exposure to digital dentistry content outside the formal curriculum, or differential access to digital technologies may also have influenced the observed association between academic year and CAD/CAM knowledge.

In this study, factors such as age group, sex, marital status, place of origin, occupation, and prior CAD/CAM training were not significantly associated with knowledge level. This may be explained by relatively homogeneous access to academic information and content provided by the curriculum, which reduces differences attributable to individual characteristics. Vlagos et al.^26^ support this interpretation, demonstrating that implementing a structured program improves student education and patient care by equipping learners with the competencies needed to operate in an evolving clinical and technological environment. It is also possible that the prior training some students received was not sufficiently in-depth or specialized to yield measurable differences.

A key strength of this study is the inclusion of dental students from campuses in both the capital and a provincial setting, capturing diverse perspectives within a uniform curriculum. This reduces confounding from program heterogeneity and supports the inference that observed differences reflect student-level factors rather than curricular variation. A further strength is the census-like approach at the selected campuses, whereby all eligible students were invited, thereby minimising sampling bias^27^. This enhances the representativeness of the findings for the institutional context and bolsters confidence in the inferences drawn. In addition, analytical methods were appropriate for a cross-sectional study, with suitable statistical tests and multivariable modelling, strengthening both the analysis and its interpretation^28^.

Among the limitations, the outcome was derived from a de novo questionnaire that underwent only an initial psychometric assessment; therefore, the findings should be interpreted as reflecting questionnaire-assessed knowledge rather than objective or externally validated competence in CAD/CAM^29^. Although content validity and test–retest reliability were satisfactory, internal consistency was moderate (Cronbach’s alpha = 0.67; 95% CI: 0.51–0.79) and did not reach the ≥ 0.80 threshold commonly recommended for instruments used in analytical studies. This degree of measurement error may have attenuated associations toward the null, thereby reducing the ability to detect weaker relationships between student characteristics and the outcome. Moreover, confirmatory factor analysis, external validity evidence (including convergent, divergent, and criterion validity), and empirically derived cut-off scores were not assessed; therefore, further validation is warranted before broader or higher-stakes applications. Although responses to the open-ended item (Q10) were independently coded by two investigators and discrepancies were resolved by consensus, an inter-rater agreement statistic (e.g., Cohen’s kappa) was not calculated; therefore, the reproducibility of this coding process could not be formally quantified. Because the outcome was derived from a self-administered questionnaire rather than from direct observation or performance-based assessment, some degree of information bias may have persisted. Although anonymity and a standardized administration procedure were used to reduce this risk, social desirability may still have led some participants to provide answers they perceived as academically appropriate, potentially overestimating the level of knowledge captured by the questionnaire. Residual confounding also cannot be excluded, as variables such as informal training, extracurricular exposure, engagement with digital dentistry resources, and access to digital technologies outside the formal curriculum were not measured. Additionally, the categorization of knowledge into “poor,” “fair,” and “good” was established a priori and used solely as operational and descriptive categories within this study, rather than as validated or competency-based thresholds. Because empirically derived cut-offs (e.g., criterion-referenced thresholds or ROC-based approaches) were not established, these categories should be interpreted with caution. Future research should establish more robust and empirically grounded cut-off points to enable more precise measurement of CAD/CAM-related knowledge. Nevertheless, the present findings provide a useful basis for subsequent studies aimed at identifying knowledge gaps and informing the planning and reinforcement of CAD/CAM training. Finally, the cross-sectional design precluded assessment of knowledge trajectories and retention over time in relation to this technology^28,30^.

## Conclusion

This study identified knowledge gaps regarding CAD/CAM among Peruvian dental students. When questionnaire scores were summarized using prespecified descriptive score groupings, most students clustered in the fair range and a smaller proportion in the poor range. Frequent misconceptions were observed, particularly concerning physical impressions, compatible materials, and the definition of the technology. Academic year was associated with membership in the poor score grouping for CAD/CAM knowledge, with third-year students showing a higher prevalence than fifth-year students, whereas demographic variables and prior training were not significantly associated. However, these findings should be interpreted cautiously given the preliminary measurement evidence and because the score groupings were used solely as operational, descriptive categories within this study rather than as validated or competency-based thresholds. Even so, these findings may help inform curricular review and the design of future studies using more robust competency-based assessments.

## Supplementary Information

Below is the link to the electronic supplementary material.

Supplementary Material 1

## Data Availability

All data analyzed during this study are available from the corresponding author on reasonable request (cesar.cayo@upsjb.edu.pe).

## Abbreviations

APR: Adjusted prevalence ratio
CAD/CAM: Computer-aided design/computer-aided manufacturing
CAL: Client access license
CI: Confidence interval
STROBE: STrengthening the Reporting of OBservational studies in Epidemiology
SPSS: Statistical package for the social sciences

## Declarations

### Generative AI and AI-assisted technologies in the writing process

During the preparation of this work the authors used ChatGPT 5.0 for proofreading of the manuscript. After using this tool, the authors reviewed and edited the content as needed and take full responsibility for the content of the publication.
