# Supplementary material for: Factors associated with the level of knowledge about CAD/CAM use among Peruvian dental students
Source: Sci Rep. 2026 Apr 29;16:19768. doi: 10.1038/s41598-026-50270-2 (PMC13314937; doi:10.1038/s41598-026-50270-2)
Supplement: Supplementary file 1 — Supplementary Material 1 [file 41598_2026_50270_MOESM1_ESM.docx]

**Supplementary material**

**Spanish questionnaire**

**Sección 1: Datos generales**

**Instrucciones:** Marque una opción por ítem. Para Edad (años) y la pregunta Q10, escriba su respuesta.

Edad (años): _______________

Sexo

• Masculino

• Femenino

Año de estudio

• Tercer año

• Cuarto año

• Quinto año

Estado civil

• Soltero

• Casado o conviviente

Lugar de origen

• Capital

• Provincia

Ocupación

• Trabaja y estudia

• Solo estudia

Capacitaciones previas en CAD/CAM

• Sí

• No

**Sección 2 – Cuestionario de conocimientos sobre el uso de CAD/CAM**

P1. ¿Es posible diseñar y fabricar una prótesis dental infalible utilizando CAD/CAM sin tener que realizar ajustes en la boca del paciente?

- Si
- No
- No sé

P2. ¿El uso del CAD/CAM en odontología elimina completamente la necesidad de impresiones físicas de la boca del paciente?

- Si
- No
- No sé

P3. ¿Es indispensable instrucción previa para asegurar un correcto uso del sistema CAD/CAM?

- Si
- No
- No sé

P4. ¿Además de los discos de zirconio podemos emplear el metal como material para utilizar la tecnología CAD/CAM?

- Si
- No
- No sé

P5. ¿Se puede realizar una prótesis total utilizando tecnología CAD/CAM?

- Si
- No
- No sé

P6. ¿Se puede realizar poste y núcleo usando tecnología CAD/CAM?

- Si
- No
- No sé

P7. ¿La tecnología de impresión tridimensional (3D) imposibilita utilizar nuevos materiales en la odontología?

- Si
- No
- No sé

P8. ¿La tecnología CAD/CAM es más precisa que los procedimientos convencionales?

- Si
- No
- No sé

P9. ¿Una restauración elaborada con tecnología CAD/CAM sería más rápida que con el método convencional?

- Si
- No
- No sé

P10. Defina el término CAD/CAM.

Respuesta: ____________________________________________________________________________

**Faithful English translation of the questionnaire**

**Section 1: General information**

**Instructions:** Please tick one option per item. For Age (years) and Q10, write your answer.

Age (years): _______________

Sex

- Male
- Female

Year of study

- Third-year
- Fourth-year
- Fifth-year

Marital status

- Single
- Married or cohabiting

Place of origin

- Capital
- Province

Occupation

- Working and studying
- Studying only

Prior CAD/CAM training

- Yes
- No

**Section 2**

**Questionnaire on knowledge of CAD/CAM use**

Q1. Is it possible to design and fabricate an infallible dental prosthesis using CAD/CAM without requiring any intraoral adjustment?

- Yes
- No
- Don’t know

Q2. Does the use of CAD/CAM in dentistry completely eliminate the need for physical impressions of the patient’s mouth?

- Yes
- No
- Don’t know

Q3. Is prior instruction indispensable to ensure the correct use of a CAD/CAM system?

- Yes
- No
- Don’t know

Q4. In addition to zirconia discs, can metal be used as a material with CAD/CAM technology?

- Yes
- No
- Don’t know

Q5. Can a complete denture be fabricated using CAD/CAM technology?

- Yes
- No
- Don’t know

Q6. Can a post and core be fabricated using CAD/CAM technology?

- Yes
- No
- Don’t know

Q7. Does three-dimensional (3D) printing preclude the use of new materials in dentistry?

- Yes
- No
- Don’t know

Q8. Is CAD/CAM technology more accurate than conventional procedures?

- Yes
- No
- Don’t know

Q9. Would a restoration fabricated using CAD/CAM be faster than with the conventional method?

- Yes
- No
- Don’t know

Q10. Define the term CAD/CAM.

Response: ___________________________________________________________________
